# A Comparative Analysis of Conventional and Chat-Generative Pre-trained Transformer-Assisted Teaching Methods in Undergraduate Dental Education

**DOI:** 10.7759/cureus.60006

**Published:** 2024-05-09

**Authors:** Amrita P Bhatia, Apurva Lambat, Teerthesh Jain

**Affiliations:** 1 Prosthodontics, Shri. Yashwantrao Chavan Memorial Medical and Rural Development Foundation's Dental College, Ahmednagar, IND; 2 General Dentistry, Affordable Dentures and Implants, Indianapolis, USA

**Keywords:** dental curriculum, chatgpt, education, dental students, conventional teaching

## Abstract

Introduction: In the present era, individuals have the ability to improve their study organization, attendance in classes, and use of mnemonics via the utilization of contemporary technology. The use of the open AI-based application Chat Generative Pre-Trained Transformer (ChatGPT) in dentistry is a developing domain, and the integration of this technology into dental education relies on the accessibility and efficacy of AI technology, as well as the readiness of institutions to adopt it. Furthermore, it is crucial to contemplate the possible ethical ramifications associated with the utilization of AI in the field of dentistry, as well as the need for dental practitioners to have adequate training in its use. In order to include the Chat Generative Pre-Trained Transformer in the dentistry curriculum, a thorough evaluation and consultation with field specialists would be necessary. This study aimed to determine whether the Chat Generative Pre-Trained Transformer is more effective than conventional teaching methods in teaching undergraduate dental students.

Method: Comparative research was conducted at Shri. Yashwantrao Chavan Memorial Medical and Rural Development Foundation's Dental College, Ahmednagar. Computer-generated random numbers were used to divide 100 students into two groups. Each group consists of 50 students. A didactic lecture was given using PowerPoint (Redmond, WA: Microsoft Corp.) for both groups. Group A was given textbooks to read and Group B used the Chat Generative Pre-Trained Transformer. An online questionnaire using Google Forms (Menlo Park, CA: Google LLC), which had been pre-validated, was sent via email to both groups. The pre- and post-test scores are then compared using the t-test.

Result: The calculated t-value is 12.263 (at 81 degrees of freedom) and the p-value is 0.000, which is less than 0.01. Therefore, the null hypothesis is rejected, and it is concluded that conventional method scores and ChatGPT method scores for the post-test have a high significant difference. Also, it is observed that the mean scores for the conventional method are higher than the mean scores for the ChatGPT method for the post-test.

Conclusion: It has been concluded from the study that traditional teaching methods are more effective for learning than understanding ChatGPT.

## Introduction

Education is an ongoing process of passing information from one generation to the next. This is vital to dentistry as well. Various pedagogical approaches exist that facilitate students' acquisition of subject knowledge and their ability to apply concepts in future contexts. Lectures have traditionally been the predominant method of instruction and the acquisition of knowledge. An efficiently structured lecture is among the most efficient methods of conveying knowledge from diverse sources [1]. Baxi et al. stated that the primary objective of the lecture is to facilitate the student's comprehension and retention of the subject matter presented by the instructor. Utilizing audiovisual tools may improve the quality of the lecture. The chalkboard has been a widely used educational instrument since the 18th century. AI has emerged as a significant approach in education because of its ability to provide education at reduced expenses, offer accessibility regardless of location or time, and overcome several conventional educational obstacles, such as limited classroom availability and instructor scarcity. In the present era, individuals have the ability to improve their study organization, attendance in classes, and use of mnemonics via the utilization of contemporary technology [2]. Research on the creation of AI-powered instruments for the automated identification of dental caries and other oral disorders has been ongoing since the 1990s [3]. The use of artificial intelligence (AI) to assist in dentistry diagnosis and treatment planning is a relatively new and ongoing area of research that is still in its early phases of advancement. It is a relatively new development to integrate artificial intelligence into dentistry, and its acceptance is dependent on the individual AI applications being considered. Artificial intelligence (AI) has the potential to be used in dentistry in many ways, such as automating radiography and CT scan analysis through the use of machine learning algorithms [3]. Research on this topic has been conducted since the 1980s. The use of ChatGPT in dentistry is a developing domain, and the integration of this technology into dental education relies on the accessibility and efficacy of AI technology, as well as the readiness of institutions to adopt it. In order to include the Chat Generative Pre-Trained Transformer in dental curriculum, a thorough evaluation and consultation with field specialists would be necessary [4]. The demand from dentists, students, and clinical practitioners will be critical, given the obvious benefits that the Chat Generative Pre-Trained Transformer will deliver to patients. The quick adaptation of students to using AI writing tools, such as the Chat Generative Pre-Trained Transformer, will force instructors to reevaluate their teaching techniques and student assessments. This technology has the potential to serve as a double-edged sword, enabling students to cheat while also acting as a valuable teaching assistant and fostering creativity. However, both students and instructors in this period of many teaching methods have consistently recognized the significance of a well-delivered lecture using verbal communication and a chalkboard [5]. There is a lack of literature on the Chat Generative Pre-Trained Transformer on the efficacy of using in dentistry. This research assesses the acquisition of information by comparing traditional and Chat Generative Pre-Trained Transformer methodologies. This study aimed to determine whether conventional teaching methods or chat GPT are more effective at teaching the subject.

## Materials and methods

**Figure 1:**
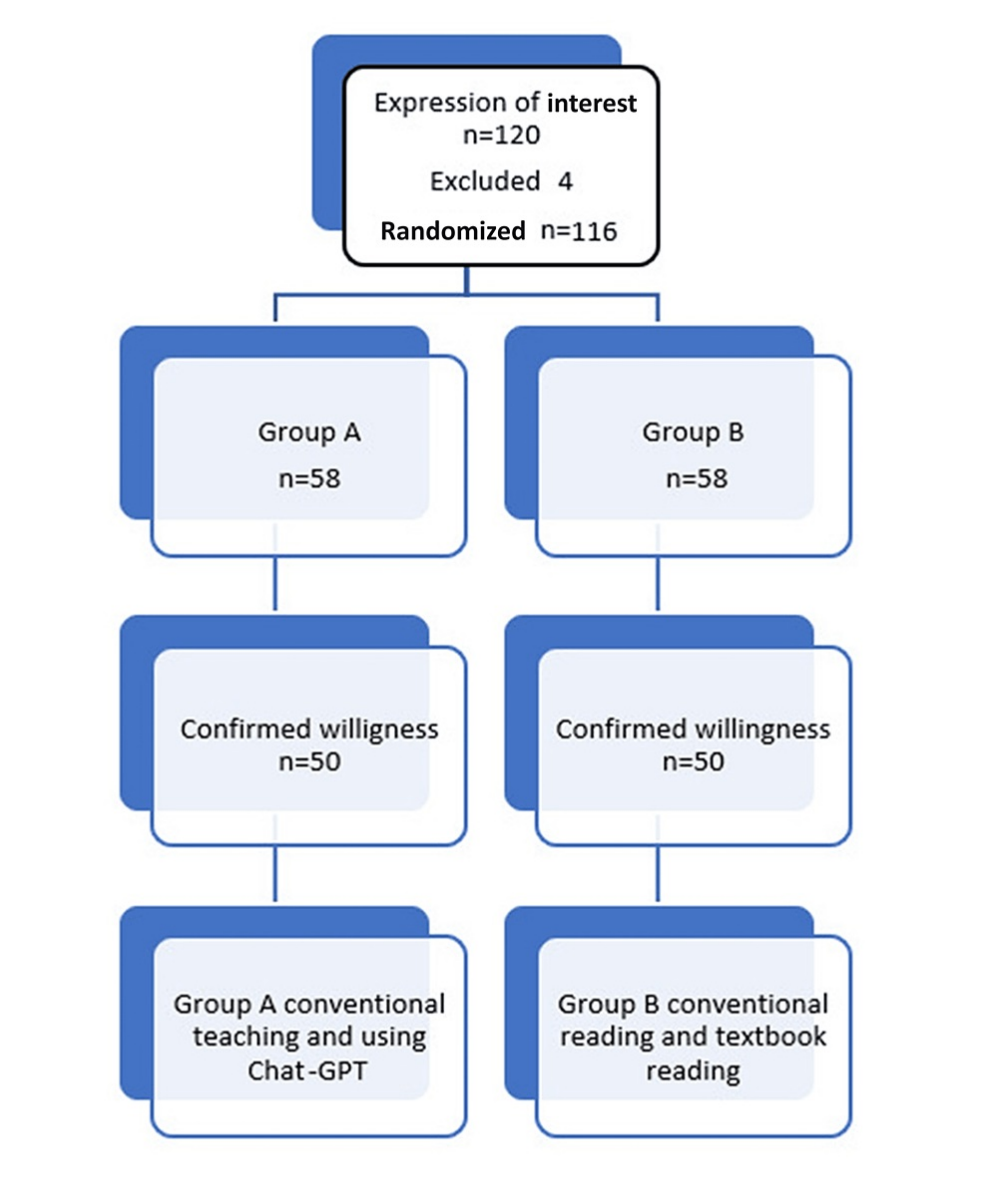
Participant allocation flow diagram. ChatGPT: Chat Generative Pre-Trained Transformer

Comparative research was conducted at Shri. Yashwantrao Chavan Memorial Medical and Rural Development Foundation's Dental College, Ahmednagar, India. Computer-generated random numbers were used to divide 100 students into two groups. Each group consists of 50 students. A didiactic lecture was taken using PowerPoint (Redmond, WA: Microsoft Corp.) for both groups. Group A was given textbooks to read and Group B used the Chat Generative Pre-Trained Transformer (Figure 1). An online questionnaire using Google Forms (Menlo Park, CA: Google LLC), which had been pre-validated, was sent via email to 100 undergraduate students at Shri. Yashwantrao Chavan Memorial Medical and Rural Development Foundation's Dental College and Hospital, Ahmednagar. The pre- and post-test scores are then compared using the t-test. An online informed consent was taken from every participant prior to the lecture for participating in the study. The questionnaire was validated by specialists, and then a pilot study was done with a sample size of 25. Further results were calculated and tabulated, and the final validation was done by a statistician with ethical approval from the institution (approval number: YCDC/IES-IRC /30/2023-24).

Sample size calculation

The formula used for calculating the sample size is as follows.

$n = \frac{{Z_{\alpha }}^{2}p(1-p)}{e^{2}}$

Here, n represents the sample size, Zα is the standardized normal value at the desired confidence level (here, 95% confidence level is used, which corresponds to Zα=1.96), p is the estimated proportion (here assumed to be 0.5 because the true proportion is unknown), and e is the margin of error, which indicates the maximum allowable difference between the sample estimate and the true population parameter.

Using the below formula, we can calculate the sample size.

$n = \frac{(1.96)^{2}0.5(1-0.5)}{(0.1)^{2}}=\frac{3.8416\times 0.5\times0.5}{0.01}=96.04\simeq 96$

Rounding to the nearest whole number, the calculated sample size for a 95% confidence interval is approximately 100.

Method of data analysis

Descriptive qualitative data will be expressed in frequencies and percentages. Since the variables are categorical, their association will be tested using the chi-square test, and the proportion of two variables will be assessed using the binomial test. This study aimed to determine whether the Chat Generative Pre-Trained Transformer is more effective than conventional teaching methods in teaching undergraduate dental students. Pre- and post-test questionnaires were issued. The pre-test questions include fundamental inquiries about the anatomical landmarks of the maxilla and mandible. As with the pre-test, the post-test questionnaire has the same questions, but it is not in the same order and includes some image-based questions, as well as questions about the benefits of artificial intelligence in education. One hundred students were administered a pre-validated pre-test questionnaire. After this, two groups were randomly formed, with one group consisting of 50 students. Using PowerPoint presentations and whiteboards, all students were taught through traditional lectures. Topics covered included "the anatomical landmarks of the maxilla and mandible." The lecture was followed by the formation of two random groups as follows: the first group was given textbooks to read about the same subject as the lecture, and the second group was allowed to use ChatGPT to learn both the same topic. Post-test questionnaires were provided to both groups. The second group of students was given instructions to install the Chat Generative Pre-Trained Transformer application on their mobile devices and thereafter use it to study anatomical landmarks. Both groups were given 30 minutes for learning. Subsequently, the post-test questionnaire was provided to the same group of students. Analyzed using IBM SPSS Statistics version 20 software (Armonk, NY: IBM Corp.), statistical analysis was conducted both between and within the groups. The participants' identities were kept confidential in order to mitigate prejudice. All of the questions have been combined and converted into a single score because the information that has been converted is in numerical form. The pre- and post-test scores are then compared using the t-test. Moreover, the data collection was scheduled for a period of two weeks and after that statistical analysis was performed.

## Results

The null hypothesis (there is no significant difference between pre-test scores and post-test scores) is rejected and it is concluded that pre-test and post-test scores for the conventional teaching methods have significant differences. Hence, it can be said that there is a substantial disparity in scores between the pre-test and post-test for the conventional teaching methods, as described in Table 1. In addition, it has been observed that the mean scores for the post-test are higher than the mean scores for the pre-test. Since, the calculated t-value is 12.263 (at 81 degrees of freedom) and p-value is 0.000, which is less than 0.01, the null hypothesis (there is no significant difference between conventional method scores and ChatGPT method scores for post-test) is rejected, and it is concluded that conventional method scores and ChatGPT method scores for the post-test have a high significant difference. Also, It is observed that the mean scores for the conventional method are higher than the mean scores for the ChatGPT method for the post-test (Table 2). It may be inferred that there is a significant difference between the scores obtained using the conventional approach and the scores obtained using the ChatGPT method for the post-test. Furthermore, it has been noted that the average scores for the traditional approach are higher than the average scores for the ChatGPT method in the post-test (Table 3). Therefore, it may be inferred that traditional teaching methods are more effective for learning than understanding ChatGPT.

**Table 1:** Calculation of content validity ratio for pre-validation of questionnaire. $\textrm{Content validity ratio} =\frac{n_{e}-\frac{N}{2}}{\frac{N}{2}}$ Here, ne is the number of subject matter experts indicating “essential” and N is the total number of SME panelists. $\textrm{Average content validity ratio} = \frac{1}{10}\times \frac{n_{e}-\frac{N}{2}}{\frac{N}{2}} = 0.52$ SME: subject matter experts

Question no.	n_e_	N	Content validity ratio
1	3	5	0.2
2	4	5	0.6
3	5	5	1
4	4	5	0.6
5	3	5	0.2
6	2	5	-0.2
7	4	5	0.6
8	5	5	1
9	3	5	0.2
10	5	5	1

**Table 2:** Comparison between conventional teaching and after using ChatGPT. *P-value less than 0.001 is considered statistically significant. All the data is arranged into 0s and 1s, with "0" indicating an incorrect answer and "1" indicating a correct answer, in order to determine the overall finding. All questions have been combined and converted into a single score because the information converted is in numerical form. The pre- and post-test scores are then compared using the t-test, and the results are presented in the table. ChatGPT: Chat Generative Pre-Trained Transformer

Variables		Mean	N	Std. deviation	Std. error mean	t-test	p-Value
Method	Conventional	3.57	82	0.498	0.055	12.263	0.000*
	ChatGPT	1.7	82	1.33	0.147		

**Table 3:** Association between pre-test and post-test responses for the conventional method. *Levels of significance for three of the most commonly used levels. P-value less than 0.05 is considered statistically significant.

Questions	Test		Chi-square value	p-Value
	Pre	Post		
1. The primary stress-bearing area of maxillary complete denture is				
Incorrect	54 (65.85)	69 (84.15)	7.317	0.007*
Correct	28 (34.15)	13 (15.85)		
2. The mean denture-bearing area in the edentulous mandible is approximately				
Incorrect	15 (18.29)	16 (19.51)	0.040	0.842
Correct	67 (81.71)	66 (80.49)		
3. Primary stress-bearing area in mandibular edentulous ridge is				
Incorrect	23 (28.05)	32 (39.02)	2.216	0.137
Correct	59 (71.95)	50 (60.98)		
4. Denture-bearing area of the ridge is				
Incorrect	25 (30.49)	9 (10.98)	9.499	0.002*
Correct	57 (69.51)	73 (89.02)		
5. Which of the following are stress-bearing areas				
Incorrect	34 (41.46)	27 (32.93)	1.279	0.258
Correct	48 (58.54)	55 (67.07)		
6. The soft tissue area beyond the junction of the hard and soft palates on which pressure within physiological limits, can be applied by a complete denture to aid in its retention				
Incorrect	28 (34.15)	17 (20.73)	3.706	0.054
Correct	54 (65.85)	65 (79.27)		
7. These are two indentations on each side of the midline, formed by a coalescence of several mucous gland ducts				
Incorrect	62 (75.61)	49 (59.76)	4.711	0.030*
Correct	20 (24.39)	33 (40.24)		
8. It overlies the medial palatal suture, extended from the incisive papilla to the distal end of the hard palate				
Incorrect	40 (48.78)	26 (31.71)	4.97	0.026*
Correct	42 (51.22)	56 (68.29)		
9. Secondary stress-bearing area of maxilla				
Incorrect	57 (69.51)	45 (54.88)	3.734	0.053
Correct	25 (30.49)	37 (45.12)		
10. The anterior exit of the mandibular canal located on the external surface of the mandible between the first and second premolar area				
Incorrect	40 (48.78)	49 (59.76)	1.99	0.158
Correct	42 (51.22)	33 (40.24)		

## Discussion

According to our study, it has been suggested that conventional teaching and reading are better for educating students than ChatGPT. Ali et al. conducted a similar study in 2023, in which they concluded that ChatGPT has advantages and disadvantages [6]. While it can benefit students and teachers, it can also generate assignments and answers, leading to academic dishonesty. The Chat Generative Pre-Trained Transformer provides an intelligent learning platform that scaffolds students learning, adapting and personalizing learning content based on their needs. Using ChatGPT as a learning platform appears to be an acceptable option and does not raise any concerns [6]. There is always a risk of inaccurate information on the web, which is also true for ChatGPT, so users should always cross-check the information they find when in doubt. The challenges posed by innovative technologies are not new to education providers. The current generation of academics has already experienced the internet revolution. The use of powerful search engines, web-based applications such as YouTube (Menlo Park, CA: Google LLC), and digital flashcards has transformed access to information. It is imperative that students get information as fast as possible [7].

Therefore, they use Chat Generative Pre-Trained Transformer to make presentations and complete assignments. Lectures are the most often utilized teaching modality in medical and dental schools. Students believe that didactic lectures are the most effective teaching and learning medium because they allow students to participate actively. Teaching strategies that promote self-directed learning may be helpful in imparting essential information, leading to enhanced learning. Our research found that employing presentation and traditional methods to measure students’ knowledge resulted in higher post-test scores compared to pre-tests. The traditional style of teaching is more successful since it involves the instructor writing the theory and relating it to the topic [8]. Not only is understanding required in reading, but also critical and creative processing of reading materials must be done while reading. Reading is not only a process of remembering, but also a mental work process that involves aspects of critical and creative thinking. A teacher can clarify the point better. Publications and other media that highlight the institution's use of AI may have a favorable influence on student recruitment. AI's position in education and health care may assist students and teachers by piquing interest in better patient care, technology-driven therapy, and teaching in a contemporary curriculum. By concentrating on AI-driven programs and procedures, the school may serve more patients in need while also attracting students who share its beliefs.

We can accelerate the deployment of AI by recruiting people who are willing to participate in the early adoption of technology in education and healthcare. A bright future for the present professors must be guaranteed. The fear of job loss that typically comes with technology deployment may be addressed by stressing change as a stimulus for professional growth and explicitly attaching incentives to it. It demonstrates a commitment to present stakeholders and their future success by offering AI-focused continuing education and faculty training opportunities [9] Artificial intelligence may be included in dental school courses; however, full dependence on diagnosis should be limited, and further study should be undertaken to assure total dependability [10]. The proper use of AI in dentistry education goes beyond patient care and curriculum. Using AI in nonclinical administrative procedures such as admissions, evaluation, and student progress would enable more efficient use of human resources while also improving insights and prioritizing care delivery. Ethical concerns and ongoing improvement methods must be included in the algorithms and review procedures as well. Students should be exposed to the use of these tools so that they may include them in their future activities [11].

The younger generation of medical and dental professionals sees AI as a tool for collaboration in their practices. Strong interest and active participation in AI-related courses emphasize the necessity of incorporating AI education into medical curriculum to foster new healthcare practices [12]. Despite the fact that dental students had little awareness of AI, they were eager to learn more about emerging dentistry-related technologies. Additionally, participants predicted that AI will play a significant role in dentistry. Lectures, curricular courses, and scientific meetings should all be taken into account while improving dentistry students' understanding of artificial intelligence [13].

AI and other digital technologies will be used in clinical practice, regardless of specialty. It would be a failure if we did not take this chance to properly prepare our future doctors with the necessary information. We believe that tomorrow's doctors must be able to utilize digital technologies, including AI, in a way that is consistent with rational evidence-based drug usage [14].

According to our study, it has been suggested that conventional teaching and reading are better for educating students than ChatGPT. ChatGPT has advantages and disadvantages. This study is the first to investigate the impact of generative AI represented by ChatGPT and conventional teaching methods on commonly used assessments in dental education. Our results demonstrate that conventional teaching was better than using ChatGPT for learning. In the future, AI technologies will most likely be employed to gather, analyze, and organize patient-related information in order to deliver patient-centered, personalized dental care [15]. While it can benefit students and teachers, it can also generate assignments and answers, leading to academic dishonesty. Although it has some limitations, it can revolutionize virtual learning. Rather than treating it as a threat, dental educators should adjust teaching and assessments to ensure that learners benefit without resorting to academic dishonesty [16]. As Chat Generative Pre-Trained Transformer becomes more popular, students may plan assignments and deliver fairly correct solutions to a broad variety of test questions that are often utilized in the evaluation of students across the board, including dentistry undergraduates.

The Chat Generative Pre-Trained Transformer offered correct replies to the majority of knowledge-based exams using multiple choice questions (MCQs). However, it could only answer text-based queries and could not support image-based inquiries. The Chat Generative Pre-Trained Transformer's educational value is promising, and its implementation in dentistry education may offer a personalized learning experience to meet the diverse learning requirements of dental students. However, from this study, it has been noted that traditional teaching methods are more effective for learning than utilizing Chat Generative Pre-Trained Transformer. Students felt that the knowledge of Chat Generative Pre-Trained Transformer is limited and not very reliable. Images and videos were not there on the given topic on ChatGPT hence students used Google and other internet applications to search for answers. Unlike Chat Generative Pre-Trained Transformer, traditional teaching methods are confined to school hours and the availability of teachers. It’s challenging for a single teacher to cater to the individual needs of every student in a classroom. Whereas, by reading books and attending lectures, students could answer better, as all the necessary information is there in the book and many doubts can be cleared by the lecturer. But, again, if we want instant answers, this method is not appreciated. For that, we can use ChatGPT. Finding a balance between these two methods is the key. As an example, the Chat Generative Pre-Trained Transformer can be used for initial drafts and research, while traditional methods can be used for in-depth analysis and discussion. This way, the strengths of one can compensate for the weaknesses of the other, creating a more robust educational experience. The limitations of the study are that the multiple choice questions were distributed to generalize the knowledge of the students on a given topic. Instead, using written paper would have given more accurate results [16]. Also, there were many students who were unfamiliar with the Chat Generative Pre-Trained Transformer. There could have been a larger sample size to allow for a more general analysis.

## Conclusions

According to the objectives of the study, it can be concluded that students could learn better using traditional methods. This is because a lot of information can be provided by the teacher, as well as by reading textbooks. Unlike ChatGPT, which has very limited information available, the information provided is not reliable, and there are no videos or pictures of the topic available. A Chat Generative Pre-Trained Transformer is a two-edged tool that can be useful for both teachers and students. However, it can also be used to create assignments and provide answers to assessment questions, which raises questions about possible academic dishonesty and cheating. Even with their present drawbacks, generative AI applications have the power to completely transform online education. Students and educators should be educated about the limitations of Chat Generative Pre-Trained Transformer as part of curriculum design. It is vital that awareness programs are implemented to promote critical engagement and responsible use of Chat Generative Pre-Trained Transformer. The integration of ChatGPT and traditional teaching methods is more than just a trend. It is a necessity for the future. By focusing on these research directions, we can lay the groundwork for a more dynamic, inclusive, and effective educational ecosystem. This study aimed to spark further dialogue and inspire collective action among educators, policymakers, and technology enthusiasts. With future advancements in Chat Generative Pre-Trained Transformer images and videos should be incorporated for a better learning experience.
